# The disputed identity of *Pseudomonas putida* KT2440: when taxonomists rename your favorite microbe

**DOI:** 10.1128/mbio.03390-25

**Published:** 2026-01-27

**Authors:** Victor de Lorenzo, Danilo Pérez-Pantoja, Juan Luis Ramos, Pablo I. Nikel

**Affiliations:** 1 Centro Nacional de Biotecnología, Consejo Superior de Investigaciones Científicas 16379, Madrid, Spain; 2 Instituto Universitario de Investigación y Desarrollo Tecnológico (IDT), Universidad Tecnológica Metropolitana 28092, Santiago, Chile; 3 Center of Applied Ecology and Sustainability (CAPES), Santiago, Chile; 4 Consejo Superior de Investigaciones Cientificas, Estación Experimental del Zaidín 73025, Granada, Spain; 5 The Novo Nordisk Center for Biosustainability, Technical University of Denmark, Kongens 5205, Lyngby, Denmark; Georgia Institute of Technology, Atlanta, Georgia, USA

**Keywords:** *Pseudomonas putida *KT2440, taxonomic reclassification, microbial nomenclature community-based naming, *P. alloputida *species delimitation, synthetic biology

## Abstract

*Pseudomonas putida* KT2440 has long served as a reference strain in environmental microbiology, biotechnology, and synthetic biology. Its recent taxonomic reclassification as *P. alloputida*, however, exemplifies the tensions generated by top-down renaming decisions that overlook long-standing community practice. Although phylogenetic analyses suggest that KT2440 differs from the type strain of *P. putida*, the new name disrupts decades of accumulated knowledge, continuity, and shared identity built around the original designation. We argue that ever-changing taxonomic orthodoxy should not override practical utility, historical coherence, and sense of community. Given the strain’s global relevance and the insignificant acceptance of the proposed new name, we advocate for retaining the traditional species name or, if necessary, adopting an alternative solution developed through broad consultation. The case of strain KT2440 highlights the need for common sense and community involvement in microbial nomenclature, especially for iconic species and strains whose names have been part of scientific communication and practice.

## COMMENTARY

Names matter; they are not mere labels. They operate as anchors of memory**,** social coordinates**,** and references of collective identity. When a name becomes established through long-term use and broad adoption, it accumulates layers of meaning—historical events, personal associations, shared stories, and tacit agreements about how a community relates to the named object or site (1). Against this backdrop, a sudden, top-down decision to rename a place, institution, or object disrupts much more than vocabulary**;** it unsettles the everyday architecture of how people manage their personal or professional environment. This caveat is particularly evident not only when the changes affect cities, regions, streets, and public infrastructure, but also (and especially) everyday objects. Top-down renaming or reclassification often comes as arbitrary events**,** even when policymakers consider it justified. The abruptness of the change interrupts cognitive routines: new names no longer match memories, traditional signs lack connection to new designations, and habitual speech suddenly becomes incorrect (2). This friction produces an initial wave of perplexity. People struggle not only with the new name but also with the implicit demand that they personally update their linguistic habits to comply with authority. This confusion often turns into irritation**,** because renaming is rarely a neutral act: it usually carries a symbolic statement of power from those who promulgate such a change (3). Even when intentions are legitimate, the fact that they are imposed without deliberation with those affected makes the change feel like a unilateral appropriation of shared patrimony. Names, after all, are not owned by governments or committees; they are validated by communal practice. Top-down renaming thus collides with conventional wisdom that linguistic conventions belong to the users, not to those claiming authority. On this background, microbiologists often face puzzlement that taxonomists alien to their field of work decide to rename a historically used designation of species or strains that have long been adopted by the community (4–6).

In this commentary, we examine the case of *Pseudomonas putida* KT2440 as a particularly striking example among the many puzzling renaming propositions that often pop up in microbiological literature (Table 1). This bacterial strain has long held a well-established name and status within the community. Widely recognized as a model environmental bacterium with notable abilities in rhizosphere colonization and bioremediation, *P. putida* KT2440 has become an increasingly important platform for synthetic biology and metabolic engineering (7). Over the years, a substantial body of work about this species has been accumulated across multiple disciplines. The issue at stake is that strain KT2440 has not only undergone recently a taxonomic reclassification—which, in principle, may be methodologically appropriate—but has also been assigned an entirely new species name: *P. alloputida* (8). This change abandons the extensive use and continuity attached to the original designation, while introducing more confusion than clarity for hundreds of researchers who rely on the strain as the focus of their work.

**TABLE 1:** Non-exhaustive sample of iconic microbial species that have undergone conspicuous name changes

Original name	Subsequent names (chronological order)	Years
*Pseudomonas cepacia*	*Burkholderia cepacia → Ralstonia cepacia → Burkholderia cepacia*	1950s (orig.) → 1992 → 1995-96 → 1997–2000 (return to *Burkholderia*)
*Pseudomonas pseudomallei*	*Burkholderia pseudomallei* → attempts to split into genomovars	1912 (orig.) → 1992 → 2000s (proposals)
*Pseudomonas maltophilia*	*Xanthomonas maltophilia → Stenotrophomonas maltophilia*	1960s (original) → 1978 → 1983
*Pseudomonas alcaligenes*	*Alcaligenes alcaligenes → Achromobacter* spp. (various revisions)	1950s–1970s → 1980s → 1990s
*Pseudomonas syzygii*	*Ralstonia syzygii → R. solanacearum* species complex *→ R. pseudosolanacearum*	1980s → 1995 → 2014-2016 (phylotype-based restructuring)
*Hydrogenomonas eutropha*	*Alcaligenes eutrophus → Ralstonia eutropha → Wautersia eutropha → Cupriavidus necator*	1952 → 1970s → 1990s →2004
*Agrobacterium tumefaciens*	*Rhizobium radiobacter* → returned to *Agrobacterium tumefaciens* → proposals to *Allorhizobium*	1907 → 1990s → 2000s (broad rejection by the community) → 2018 (proposal)
*Agrobacterium rhizogenes*	*Rhizobium rhizogenes → Allorhizobium* (proposed)	1930s → 1990s → 2018
*Ideonella sakaiensis*	*Piscinibacter sakaiensis* (proposed)	2016 → 2023
*Corynebacterium pyogenes*	*Arcanobacterium pyogenes → Trueperella pyogenes*	Pre-1930s → 1982 → 2011
*Flavobacterium meningosepticum*	*Chryseobacterium meningosepticum → Elizabethkingia meningoseptica*	1950s → 1994 → 2005
*Anabaena* spp. (cyanobacteria)	*Dolichospermum* spp. ↔ back to *Anabaena* (dual botanical + bacterial codes)	Multiple cycles: 1900s → 2011 → 2014 → ongoing
*Nostoc/Trichormus* group	Recurring reassignments between *Nostoc, Anabaena, Trichormus*	1800s → 1990s → 2010s → ongoing
*Zygosaccharomyces pastoris*	*Pichia pastoris → Komagataella pastoris → Komagataella phaffii*	1930s → 1960s → 2005

Strain mt-2, originally identified as *Pseudomonas arvilla*, later reclassified as *P. putida* (9), is the parental strain of *P. putida* KT2440 (10). This reclassification was followed by subdivision into biovars A and B (11). Both the type strain *P. putida* ATCC 12633ᵀ (DSM 291ᵀ) and KT2440 were classified within biovar A (11). Yet, DNA–DNA hybridization (DDH) between strains KT2440 and ATCC 12633ᵀ exposed only 50.5% genomic similarity (12), considerably below the 70% generally considered for strains of the same species (13). Further phenotype analyses supported such a divergence (12). Finally, average nucleotide identity (ANI) analyses of the genome of KT2440 yielded values <91% when compared with other *P. putida* counterparts (11), indicating that the strain could belong to a distinct *Pseudomonas* species. Therefore, that strain KT2440 was not a typical *P. putida* was well known in the community, though no one raised any issue by then. However, in 2019 Keshavarz-Tohid et al. (8) re-examined 95 strains of the *P. putida* group with new criteria and proposed their split into four new species (*P. alloputida*, *P. inefficax*, *P. persica*, and *P. shirazica*). Strain KT2440 was assigned to species they named *P. alloputida* based on 76.8% (digital DDH) and 97.3% (ANI) values with Kh7 type strain (LMG 29756ᵀ) isolated from bean rhizosphere in Iran (14). As discussed before (7), this assignment is not devoid of problems. One is that Keshavarz-Tohid et al. (14) did not consider the parental mt-2 strain directly, but only the plasmid-cured derivative strain KT2440. When GGDC (15) and JSpeciesWS (16) are used to reanalyze the data with strain mt-2, digital DDH (76.8%) and ANI (96.6%) values move away with respect to *P. alloputida*. A second issue is that currently available genomic sequence of the LMG 29756ᵀ strain is widely fragmented across 429 contigs (accession OLKK01000000) and therefore final values of all these parameters should be refined considering a complete assembly. But perhaps the most frustrating side of this whole renaming affair is the name *alloputida*. The etymology of the term derived from *allos* (Greek: “other”), thereby branding KT2440 as “another *putida*” (8). This descriptor hardly expresses the exceptional metabolic nature of this strain, which has become the best-known representative of the non-clinical *Pseudomonas* species (7). It is true that Pseudomonads is one of the bacterial groups that are more frequently subject to taxonomic turbulences (17, 18), in particular the *putida-fluorescens* group (19, 20). Yet given the huge body of seminal work, biotechnological importance, and cosmopolitan nature attributed to *P. putida* KT2440, we argue that either a recognizable name or, alternatively, a more descriptive and meaningful species designation—one that reflects these well-established traits—would provide a better fit for both its practical relevance and its long scientific trajectory than the generic and uninformative label *alloputida*.

The simplest way to address this issue is to retain the traditional designation (*P. putida*), even if it no longer sides perfectly with the ever-changing taxonomic orthodoxy of the moment. Chemistry offers countless examples of molecules that coexist under both their historical names and their formal IUPAC nomenclature—without causing outrage or confusion (21). Biology is similar in this regard: many central proteins and genes continue to be known by long-standing, non-systematic names (such as IHF, Crp, Lrp, Crc), whose historical significance has resisted even the most logical attempts at renaming (22, 23). Another option would be to designate the strain as a subspecies while preserving *putida* as the species name. If, however, a name change is considered unavoidable, the most convenient, respectful, and appropriate approach would be to consult the broader research community first—ideally at an international meeting dedicated to this microorganism, where consensus could be sought with experts. An international conference on *Pseudomonas*, which will celebrate its 40th anniversary in 2026, is held every 2 years (https://pseudomonas.io).

We recognize the substantial practical challenge of reverting or modifying a name once the International Committee on Systematics of Prokaryotes (https://www.the-icsp.org) has issued an official, top-down change at the request of taxonomists who may have no working relationship with the organism. Nevertheless, we argue that common sense and support by the research community should take precedence over the name-changing drive of those who rebrand bacteria only considering systematics issues without engaging with them directly (24). Furthermore, our request from the *Pseudomonas* community is not unprecedented. A recent meta-analysis of *Escherichia coli* genomes identified 14 distinct phylogroups, two of which correspond to *Shigella* (25). For historical and medical reasons, *Shigella* strains have preserved a separate genus name, even though it is well-known that they are essentially subspecies of *E. coli* (26).

Maintenance of long-used names—for *P. putida* KT2440 and many other iconic microbial strains—has also a pragmatic dimension. Renaming incurs costs—economic, organizational, and even emotional. Researchers must adapt to the new designation, update papers, reconfigure literature-searching systems, or simply live with a prolonged period of double naming. Each of these frictions reinforces the perception that the potential benefits of the new name have little value for the community, while the inconvenience is personal and immediate. Microbiologists who have been working with a given strain for a long time may feel that their historical references are being overwritten or that familiar landmarks are being reframed with alien taxonomic narratives. Resistance to change, therefore, becomes not merely a complaint about a new name but a defense of the community’s right to participate in meaning-making. When renaming emerges from broad consultation, gradual adoption, or grassroots initiative, it can be embraced as a collective act of renewal. But when implemented suddenly from above, renaming is experienced as an intrusion: a reminder that language, despite being good for their users, can be appropriated and reshaped top-down with little regard for the social life of words.

This tension—between technical committees and what could be called communal linguistic sovereignty—is what makes top-down renaming uniquely prone to perplexity, irritation, and considerable resistance: according to PubMed at this time, since the proposal of the new species in 2019, no more than one dozen publications have adopted the name *P. alloputida* for strain KT2440, whereas more than 500 scientific papers published during the same period continue to refer to the strain as *P. putida*. This bears witness to the lack of fervor of the community for the new nomenclature. We therefore vindicate that common sense and practical considerations, rather than ever-shifting taxonomic trends, should guide the retention of the name *P. putida* KT2440, which underpins its wide adoption across scientific fields.
